# Exploring the impact of ESG controversies on private hospital performance in China

**DOI:** 10.1097/MD.0000000000049332

**Published:** 2026-06-12

**Authors:** Huanling Yang, Jian Xu

**Affiliations:** a School of Economics and Management, Qingdao Agricultural University, Qingdao, China.

**Keywords:** ESG controversies, hospital performance, private hospital

## Abstract

This study aims to investigate the impact of environmental, social, and governance (ESG) controversies on the performance of private hospitals in China during 2012 to 2023. ESG controversies are measured by the number of ESG controversial issues, private hospital performance is measured by return on assets and return on equity, and fixed effects models are employed. The empirical results reveal that ESG controversies have a negative impact on private hospital performance. This finding is still robust after a series of robustness checks. In addition, heterogeneous analysis shows that this impact is more prominent in hospitals that carry out digital transformation, before and after COVID-19, and in China’s eastern regions. This study will help hospital managers to improve ESG performance and hospital performance through avoiding controversial issues in today’s competitive environment.

## 1. Introduction

In recent years, the impact of environmental, social, and governance (ESG) controversies on organizational performance has garnered significant attention from academics, practitioners, and policymakers.^[1]^ ESG controversies range from environmental disasters to labor rights violations and governance failures.^[2,3]^ These factors can have detrimental effects on an organization’s long-term success and sustainability.^[4,5]^ However, despite the growing emphasis on ESG performance, the specific impact of ESG controversies on private hospital performance in China remains an underexplored area.

The study of ESG controversies in the healthcare sector, particularly in private hospitals, is of paramount importance due to the sensitive nature of healthcare services and the trust placed by patients and society in these institutions. Private hospitals in China have been expanding rapidly in recent decades, contributing significantly to the country’s healthcare system.^[6,7]^ However, with this growth, they have also faced numerous challenges, including those related to ESG issues.^[8]^ Understanding how ESG controversies affect private hospital performance can help managers develop strategies to mitigate these effects and enhance their ESG performance and profitability.

The primary purpose of this study is to investigate the impact of ESG controversial issues on the performance of Chinese private hospitals. Specifically, the study seeks to analyze whether this impact differs among private hospitals with various levels of digital transformation, before, during, and after the COVID-19 pandemic, and across different regions. ESG controversies are measured by the number of ESG controversial issues, and private hospital performance is measured by return on assets (ROA) and return on equity (ROE). This comprehensive analysis will allow for a deeper understanding of the effect of ESG controversies on private hospital performance.

The study makes the following contributions to the literature. First, this is the first study to examine the impact of ESG controversial issues on the performance of Chinese private hospitals, which enriches the existing literature on ESG research. Second, we examine whether this impact is different in private hospitals with different levels of digital transformation, before, during, and after COVID-19, and in different regions. This can lead to a deeper understanding of the negative impact of ESG controversies. Finally, this study can help hospital managers to make relevant strategies to avoid the occurrence of ESG controversies in order to improve their ESG performance and profitability.

## 2. Methods

### 2.1. Sample

Our research sample includes private hospitals listed on the Shanghai and Shenzhen stock exchanges from 2012 to 2023. We exclude hospitals with incomplete data, delisted hospitals, and special treatment hospitals. Finally, 13 private hospitals with 127 observations are left for further analysis. ESG controversies are manually collected from the Qinglv ESG database. The database covers over 100 types of ESG controversy incidents, comprehensively involving key aspects of environment, society, and governance, which can help stakeholders effectively identify and manage these risks. Other data are sourced from the CSMAR database. In conducting this study, we ensure adherence to ethical standards and guidelines. Since our research primarily involves the analysis of secondary data collected from the CSMAR database, focusing on financial and operational metrics of private hospitals, and does not involve any direct interaction with human subjects or the use of personally identifiable information, ethics committee or institutional review board approval is deemed unnecessary for this study.

### 2.2. Variable definition

[1] Dependent variable. Referring to Chen et al^[6]^ and Wang and Xu,^[7]^ hospital performance is measured by ROA. In the robustness check, ROE is used as another proxy for hospital performance. These 2 indicators are critical financial metrics for evaluating organizational performance due to their ability to reflect operational efficiency and financial health.^[9]^ Hospitals should monitor both to balance operational efficiency with financial sustainability, ensuring long-term service delivery and stakeholder confidence.
[2] Independent variable. At present, there are 2 methods to measure ESG controversies (ESGCON). The first method is based on the ESG controversies scores from the LSEG Workspace (previously known as the Refinitiv Eikon database), which is widely used by most scholars.^[1,10–13]^ The second method is to use the number of ESG controversial issues^.[14,15]^ In this study, we use the second method to measure ESG controversies.
[3] Control variables. Guided by previous literature,^[1,16]^ hospital size, debt ratio, current ratio, board size, and gross domestic product are chosen in this study. Table 1 shows the definition of all variables.

**Table 1:** Variable definition.

Variable	Symbol	Measurement
Return on assets	ROA	Net income/total assets
Return on equity	ROE	Net income/total shareholders’ equity
ESG controversies	ESGCON	The number of ESG controversial issues
Hospital size	SIZE	Natural logarithm of total assets
Debt ratio	LEV	Total liabilities/total assets
Current ratio	CR	Current assets/current liabilities
Board size	BOARD	The number of board members
Gross domestic product	GDP	Growth rate of gross domestic product

BOARD = board size, CR = current ratio, ESGCON = ESG controversies, GDP = gross domestic product, LEV = debt ratio, ROA = return on assets, ROE = return on equity, SIZE = hospital size.

### 2.3. Model specification

Model (1) is used to examine the impact of ESG controversies on hospital performance. The 2-way fixed effects model is constructed as follows:


$$\begin{array}{cc} & ROA_{it}=\beta_{0}+\beta_{1}ESGCON_{it}+\beta_{3}SIZE_{it}+\beta_{4}LEV_{it}\\ & +\beta_{5}CR_{it}+\beta_{6}BOARD_{it}+\beta_{7}GDP_{it}+YEAR+FIRM+\varepsilon_{it} \end{array}$$

where i is the firm; t is the year; ß is the parameter; YEAR represents year fixed effects; FIRM represents firm fixed effects; ε is the error term.

## 3. Results

### 3.1. Descriptive statistics

Table 2 shows the descriptive statistics. ROA has a mean value of 0.0144 with a minimum of −0.5087 and a maximum of 0.3070, suggesting that Chinese private hospitals need to improve their profitability. The mean value of ESGCON is 0.3543. In addition, we find that most private hospitals have experienced ESG controversial issues in recent years. SIZE has a mean value of 21.8876. The mean value of LEV indicates that private hospitals maintain a good capital structure. Current ratio has a mean value of 2.0752, and BOARD has a mean value of 8.4094.

**Table 2:** Descriptive statistics.

Variable	N	Mean	Median	Maximum	Minimum	Standard deviation
ROA	127	0.0144	0.0290	0.3070	−0.5087	0.1165
ESGCON	127	0.3543	0	6	0	0.9721
SIZE	127	21.8876	21.7015	24.1307	20.0435	0.9128
LEV	127	0.4285	0.3874	0.9901	0.0332	0.2283
CR	127	2.0752	1.3990	27.5103	0.2729	3.0718
BOARD	127	8.4094	9	14	5	1.8574
GDP	127	0.0619	0.0680	0.0840	0.0220	0.0188

BOARD = board size, CR = current ratio, ESGCON = ESG controversies, GDP = gross domestic product, LEV = debt ratio, ROA = return on assets, SIZE = hospital size.

### 3.2. Correlation analysis

Table 3 shows the results of correlation analysis. ESGCON, LEV, and BOARD are negatively correlated with ROA. Additionally, we calculate the values of the variance inflation factor, and all of them are <5, which suggests that there is no serious multicollinearity problem.

**Table 3:** Correlation matrix.

Variable	ROA	ESGCON	SIZE	LEV	CR	BOARD	GDP
ROA	1	–	–	–	–	–	–
ESGCON	−0.313***	1	–	–	–	–	–
SIZE	0.061	0.142	1	–	–	–	–
LEV	−0.351***	−0.075	0.238***	1	–	–	–
CR	0.130	−0.016	−0.198**	−0.496***	1	–	–
BOARD	−0.177**	−0.063	0.118	0.112	0.183**	1	–
GDP	0.119	−0.197**	−0.292***	−0.144	0.054	−0.043	1

BOARD = board size, CR = current ratio, ESGCON = ESG controversies, GDP = gross domestic product, LEV = debt ratio, ROA = return on assets, SIZE = hospital size.

** *P* < .05.

*** *P* < .01.

### 3.3. Regression results

Table 4 shows the results of regression analysis. In column (1) of Table 4, the model excludes control variables, and the coefficient of ESGCON is negative and significant (β = −0.038, *t* = −2.95). In column (3), after adding control variables, firm fixed effects, and year fixed effects, the coefficient of ESGCON is still negative and significant (β = −0.039, *t* = −6.17)., which suggests that the occurrence of ESG controversies leads to a decrease in private hospital performance. In addition, LEV has a negative and significant impact on ROA.

**Table 4:** Regression results of model (1).

Variable	(1)	(2)	(3)
Constant	0.028 (1.45)	−0.497 (−1.02)	0.102 (0.18)
ESGCON	−0.038** (−2.95)	−0.046*** (−4.39)	−0.039*** (−6.17)
SIZE	–	0.032 (1.71)	0.013 (0.66)
LEV	–	−0.210** (−2.71)	−0.333** (−2.54)
CR	–	0.000 (0.00)	−0.002 (−1.53)
BOARD	–	−0.011 (−1.54)	−0.008 (−0.84)
GDP	–	0.308 (0.66)	−1.098 (−0.47)
YEAR	No	No	Yes
FIRM	No	No	Yes
R^2^	0.0982	0.3168	0.6528

*t*-values in parentheses are based on all standard errors clustered by firm.

BOARD = board size, CR = current ratio, ESGCON = ESG controversies, FIRM = firm fixed effects, GDP = gross domestic product, LEV = debt ratio, SIZE = hospital size, YEAR = year fixed effects.

** *P* < .05.

*** *P* < .01.

### 3.4. Robustness check

Table 5 shows the results of the robustness check. First, we replace the dependent variable with ROE. In column (1) of Table 5, the coefficient of ESGCON is still negative and significant. Second, we use a dummy variable (ESGCONDUM) to replace the independent variable. It takes the value of 1 if private hospitals have ESG controversial issues, and 0 otherwise. In column (2) of Table 5, the coefficient of ESGCONDUM is still negative and significant. In addition, we add another control variable (BIGFOUR) into the model. If a company’s financial reports are audited by the Big 4 accounting companies, it gives a good sign that the company’s financial statements are reliable and transparent.^[17]^ It is equal to 1 if the hospital is audited by a Big 4 auditing company, and 0 otherwise. In column (3) of Table 5, ESGCON still has a negative impact on ROA. All the results suggest that our conclusion is robust.

**Table 5:** Regression results of robustness check.

Variable	(1)	(2)	(3)
	Replacing dependent variable	Replacing independent variable	Adding control variable
Constant	0.444 (0.29)	0.065 (0.11)	0.182 (0.30)
ESGCON	−0.079* (−1.86)	–	−0.038*** (−6.20)
ESGCONDUM	–	−0.067*** (−3.37)	–
SIZE	0.030 (0.61)	0.013 (0.66)	0.010 (0.48)
LEV	−0.879** (−2.40)	−0.313** (−2.21)	−0.334** (−2.55)
CR	−0.012 (−1.70)	−0.003 (−1.54)	−0.002 (−1.56)
BOARD	−0.011 (−0.37)	−0.006 (−0.60)	−0.008 (−0.84)
BIGFOUR	–	–	0.038 (1.10)
GDP	−5.768 (−0.70)	−0.858 (−0.36)	−1.262 (−0.53)
YEAR	Yes	Yes	Yes
FIRM	Yes	Yes	Yes
R^2^	0.4061	0.6110	0.6554

*t*-values in parentheses are based on all standard errors clustered by firm.

BIG = the existence of a Big 4 auditing company, BOARD = board size, CR = current ratio, ESGCON = ESG controversies, ESGCONDUM = ESG controversies dummy variable, FIRM = firm fixed effects, GDP = gross domestic product, LEV = debt ratio, SIZE = hospital size, YEAR = year fixed effects.

* *P* < .10.

** *P* < .05.

*** *P* < .01.

### 3.5. Heterogeneous analysis

The results of heterogeneous analysis are presented in Table 6. First, we divide the sample into hospitals with digital transformation (DIG = 1) and hospitals without digital transformation (DIG = 0). The digital transformation level of private hospitals is measured by text analysis, consistent with Wu et al^[18]^ In columns (1) and (2) of Table 6, we find that the coefficient of ESGCON is negative and significant in hospitals implementing digital transformation, while its impact is not significant in hospitals without digital transformation.

**Table 6:** Regression results of heterogeneous analysis.

Variable	(1)	(2)	(3)	(4)	(5)	(6)
	DIG = 1	DIG = 0	COVID = 1	COVID = 0	EAST = 1	EAST = 0
Constant	−0.405 (−0.63)	0.326 (0.44)	0.151 (0.06)	0.102 (0.16)	−0.288 (−0.68)	1.289 (1.47)
ESGCON	−0.025** (−2.29)	−0.055 (−1.75)	−0.030 (−1.39)	−0.043*** (−3.51)	−0.045*** (−5.90)	−0.009 (−0.85)
SIZE	0.020 (0.85)	0.053 (1.12)	−0.009 (−0.09)	0.022 (0.99)	0.024 (1.56)	−0.027 (−0.89)
LEV	−0.227** (−2.50)	−0.719** (−2.84)	0.071 (0.39)	−0.390** (−2.33)	−0.187 (−1.12)	−0.423 (−2.99)
CR	0.003 (0.21)	−0.004 (−1.48)	0.008 (0.55)	−0.005** (−2.47)	0.016 (1.04)	−0.001 (−0.61)
BOARD	0.005 (0.82)	−0.060* (−1.95)	0.026 (1.02)	−0.020* (−2.13)	−0.011* (−2.14)	−0.014 (−0.92)
GDP	1.407 (0.81)	−8.484** (−2.42)	−9.595 (−1.55)	−1.606 (−0.62)	0.137 (0.06)	−4.240 (−1.15)
YEAR	Yes	Yes	Yes	Yes	Yes	Yes
FIRM	Yes	Yes	Yes	Yes	Yes	Yes
R^2^	0.6793	0.9155	0.8377	0.6748	0.7723	0.5986

*t*-values in parentheses are based on all standard errors clustered by firm.

BOARD = board size, CR = current ratio, ESGCON = ESG controversies, FIRM = firm fixed effects, GDP = gross domestic product, LEV = debt ratio, SIZE = hospital size, YEAR = year fixed effects.

* *P* < .10.

** *P* < .05.

*** *P* < .01.

Next, we divide the sample into hospitals during the COVID-19 period (COVID = 1) and hospitals before and after the COVID-19 period (COVID = 0). The COVID-19 pandemic broke out in early 2020, and in December 2022, the National Health Commission of the People’s Republic of China announced the end of the COVID-19 pandemic.^[19]^ Therefore, COVID-19 period is from 2020 to 2022. In column (3) of Table 6, the impact of ESGCON is not significant during the COVID-19 period (2020–2022). In column (4), before and after that period, ESGCON has a negative impact on ROA.

Finally, we divide the sample into hospitals in China’s eastern regions (EAST = 1) and hospitals in China’s central and western regions (EAST = 0). In columns (5) and (6), we find that the impact of ESGCON is negative and significant in China’s eastern regions, while its impact is not significant in China’s central and western regions.

## 4. Discussion

### 4.1. Result discussion

The findings of this study contribute significantly to the understanding of the impact of ESG controversies on the performance of private hospitals in China. First, the study confirms that the occurrence of ESG controversies leads to a decrease in hospital performance, highlighting the importance of ESG management for these institutions. This finding aligns with previous research that has shown a negative correlation between ESG controversies and corporate performance.^[4,5,20–25]^ The negative impact of ESG controversies on hospital performance could be attributed to factors such as loss of patient trust, increased regulatory scrutiny, and negative media coverage.

Second, the study shows that the impact of ESG controversies is more significant in private hospitals with digital transformation. This suggests that digital transformation may exacerbate the negative effect of ESG controversies on hospital performance. A possible explanation for this finding is that digital transformation increases the transparency and accessibility of information about hospitals, making it easier for stakeholders to monitor and scrutinize their ESG performance. Consequently, hospitals with higher levels of digital transformation may be more vulnerable to the negative consequences of ESG controversies. This finding contrasts with previous research that has shown a positive relationship between digital transformation and ESG performance.^[16]^

Third, the study reveals that the impact of ESG controversies on hospital performance is more significant before and after COVID-19. This could be attributed to the fact that the COVID-19 pandemic dominated the public’s attention and concern, overshadowing other issues such as ESG controversies. Additionally, the pandemic may have prioritized healthcare services and reduced the scrutiny of hospitals’ ESG performance. However, before and after the COVID-19 period, ESG controversies have a negative impact on ROA, suggesting that the pandemic only temporarily altered the relationship between ESG controversies and hospital performance.

Finally, the study shows that the impact of ESG controversies is more significant in hospitals located in China’s eastern regions compared to those in the central and western regions. This finding could be related to the differences in economic development, regulatory environment, and stakeholder expectations between these regions. Hospitals in China’s eastern regions may face higher scrutiny and expectations from stakeholders regarding their ESG performance, making them more vulnerable to the negative consequences of ESG controversies.

### 4.2. Practical implications

The practical implications of this study are significant for various stakeholders involved in private hospitals in China. Firstly, for hospital managers, the study highlights the crucial importance of addressing ESG controversies to maintain and enhance hospital performance. Given that ESG controversies have a negative impact on hospital performance, it is imperative for private hospitals to develop and implement strategies that mitigate the occurrence of such controversies, especially in the era of digital transformation. This may involve strengthening governance structures, enhancing transparency in reporting, and fostering a culture of responsibility and sustainability within private hospitals.

Secondly, hospital managers in China’s eastern regions, where ESG scrutiny is more stringent, should adopt more rigorous ESG management practices to mitigate potential controversies. In contrast, hospitals in central and western regions, while currently less affected, should still remain vigilant and proactively build ESG resilience to prepare for future increases in stakeholder expectations.

Finally, the study provides valuable insights for policymakers and regulators in the healthcare sector. By recognizing the negative consequences of ESG controversies on hospital performance, policymakers can develop and enforce regulations that promote responsible and sustainable practices among private hospitals. This, in turn, can contribute to the overall improvement of healthcare services and patient outcomes in China.

### 4.3. Limitations and future directions

This study has some limitations that need to be addressed in future research. The sample size is relatively small, and the study covers a limited time period. Future studies could expand the sample size and time horizon. Additionally, the study focuses only on private hospitals in China, and the findings may not be generalizable to other types of hospitals or countries. Further research could explore the impact of ESG controversies on hospital performance in different contexts and identify potential mitigating factors that could reduce their negative effects.

## 5. Conclusions

The primary purpose of this study is to explore the impact of ESG controversies on the performance of private hospitals in China. Using a sample of listed private hospitals, we conducted an empirical analysis to investigate this relationship. The main conclusions are summarized as follows. First, the occurrence of ESG controversies reduces private hospital performance. Second, this impact is more prominent in hospitals that carry out digital transformation, before and after COVID-19, and in China’s eastern regions. This study provides valuable insights into the relationship between ESG controversies and private hospital performance in China and offers practical implications for hospital managers.

## Author contributions

**Conceptualization:** Huanling Yang, Jian Xu.

**Data curation:** Jian Xu.

**Formal analysis:** Jian Xu.

**Investigation:** Huanling Yang.

**Methodology:** Jian Xu.

**Resources:** Jian Xu.

**Software:** Jian Xu.

**Supervision:** Huanling Yang.

**Validation:** Huanling Yang.

**Visualization:** Huanling Yang.

**Writing – original draft:** Huanling Yang, Jian Xu.

**Writing – review & editing:** Jian Xu.
